# Monitoring the resilience of a no-take marine reserve to a range extending species using benthic imagery

**DOI:** 10.1371/journal.pone.0237257

**Published:** 2020-08-12

**Authors:** Nicholas R. Perkins, Geoffrey R. Hosack, Scott D. Foster, Jacquomo Monk, Neville S. Barrett

**Affiliations:** 1 Institute of Marine and Antarctic Studies, Hobart, Tasmania, Australia; 2 Data 61, CSIRO, Hobart, Tasmania, Australia; Secretariat of the Pacific Community, NEW CALEDONIA

## Abstract

Global climate change is driving the redistribution of marine species and thereby potentially restructuring endemic communities. Understanding how localised conservation measures such as protection from additional human pressures can confer resilience to ecosystems is therefore an important area of research. Here, we examine the resilience of a no-take marine reserve (NTR) to the establishment of urchin barrens habitat. The barrens habitat is created through overgrazing of kelp by an invading urchin species that is expanding its range within a hotspot of rapid climate change. In our study region, a multi-year monitoring program provides a unique time-series of benthic imagery collected by an Autonomous Underwater Vehicle (AUV) within an NTR and nearby reference areas. We use a Bayesian hierarchical spatio-temporal modelling approach to estimate whether the NTR is associated with reduced formation of urchin barrens, and thereby enhances local resilience. Our approach controls for the important environmental covariates of depth and habitat complexity (quantified as rugosity derived from multibeam sonar mapping), as well as spatial and temporal dependence. We find evidence for the NTR conferring resilience with a strong reserve effect that suggests improved resistance to the establishment of barrens. However, we find a concerning and consistent trajectory of increasing barrens cover in both the reference areas and the NTR, with the odds of barrens increasing by approximately 32% per year. Thus, whereas the reserve is demonstrating resilience to the initial establishment of barrens, there is currently no evidence of recovery once barrens are established. We also find that depth and rugosity covariates derived from multibeam mapping provide useful predictors for barrens occurrence. These results have important management implications as they demonstrate: (i) the importance of monitoring programs to inform adaptive management; (ii) that NTRs provide a potential local conservation management tool under climate change impacts, and (iii) that technologies such as AUVs and multibeam mapping can be harnessed to inform regional decision-making. Continuation of the current monitoring program is required to assess whether the NTR can provide long term protection from a phase shift that replaces kelp with urchin barrens.

## Introduction

Distributional changes of marine species associated with climate change are being documented worldwide (e.g. [1, 2]). In cases where newly arriving species play a dominant ecological role, dramatic ecosystem changes or “phase shifts” may occur with major impacts on endemic communities. Understanding how conservation measures may confer resilience, that is, the ability to resist and recover from disturbances [3, 4], is therefore a key conservation priority in the face of climate change [5]. Conservation measures that may confer resilience, such as the protection of habitats or species from additional human pressures [6], are typically legislated and managed on smaller scales than those over which climate change impacts occur. Therefore, studies that examine the efficacy of localised conservation measures in protecting habitats and biodiversity are required.

No-take reserves (NTRs) are areas where fishing pressure is reduced/removed. NTRs provide researchers with a means of examining the resilience of ecosystems that may have more “intact” communities compared to fished areas. The greater abundance and size of predators in some NTRs has been linked to increased community stability when compared to adjacent fished areas (e.g. [7–10]) as well as possible dampening of marine pest outbreaks [11]. Ecological theory developed from invasion ecology suggests that that the top-down control of higher trophic level predators can aid in reducing the establishment and persistence of invasive or range extending species (e.g., [11–14]). However, the number of empirical studies examining the effect of NTRs on local ecosystem resilience to climate-driven invasive species impacts is limited by the technical difficulties associated with acquiring high-quality time-series monitoring data in the marine environment.

Here, autonomously acquired marine imagery is coupled with spatio-temporal statistical analyses to assess how an NTR mediates local ecosystem resilience in a regional hotspot of global climate warming. The marine region of south east Australia, and in particular the east coast of Tasmania, has been identified as having warming above the global average [15, 16]. The strengthening of the East Australian Current (EAC) has resulted in increased ocean temperatures and an extended poleward penetration of the current over the last 60 years. This EAC extension has coincided with the arrival of many new species from sub-tropical latitudes into this temperate mid-latitude region [17]. Perhaps the most ecologically important new arrival is the long-spined sea urchin *Centrostephanus rodgersii* (Agassiz). The first records of this species in northern Tasmania were recorded in the late 1970s, and sightings have been increasingly moving poleward through time [18]. This urchin species is an ecosystem engineer, capable of transforming productive kelp beds and invertebrate covered reefs into bare-rock barrens with major impacts on biodiversity and flow-on effects for economically important rock lobster and abalone fisheries [19].

Dive-based and towed video surveys of urchin densities and barrens habitat on the east coast of Tasmania have highlighted concerning trends over the last 15 years, with an estimated increase in *C*. *rodgersii* numbers of approximately 50% and an overall four-fold increase in barrens habitat between 2001 and 2016 [18, 20]. However, Tasmanian NTRs may offer resilience to barrens formation and expansion due to the higher density of predators able to control urchin densities [7, 21, 22]. The main potential predator of *C*. *rodgersii* is the rock lobster *Jasus edwardsii*, a species that is the target of commercial and recreational fisheries. Higher densities of lobsters inside Tasmanian NTRs have been shown to result in three to seven times higher predation rates, depending on habitat and potential refuge for urchins [21]. Monitoring the impacts of *C*. *rodgersii* across its extended range is an important issue as effective conservation and fisheries management requires an understanding of: (I) the spatial distribution of urchins and barrens habitat, (ii) the extent and rate of expansion of barrens habitat through time, and (iii), whether the spread of barrens may be mitigated by NTR establishment.

Long term monitoring of urchin benthic habitat over large spatial extents is a challenging problem. The use of imagery as a monitoring tool for the marine benthos has been increasing over recent decades. Sampling platforms such as Autonomous Underwater Vehicles (AUVs), towed and drop camera systems and Remotely Operated Vehicles (ROVs) have allowed collection of large amounts of data over greater areas and deeper depths than have been traditionally surveyed using diver-based approaches [23, 24]. Imagery or video footage from these platforms can typically be geolocated, allowing for spatial components to be incorporated into subsequent analyses; including the co-location of observations with mapping products such as multibeam sonar (e.g., [25]) and the analysis of spatial patterns in distributions of target species (e.g., [26]). NTR monitoring programs utilising these platforms include Australia’s Integrated Marine Observing System (IMOS) AUV program and California’s Marine Protected Area ROV monitoring program. These programs now have data spanning over a decade in some reserves. As these programs are located across regions experiencing climate change impacts, an opportunity exists to utilise the available monitoring data to compare NTRs and adjacent fished areas through time.

Here we use a Bayesian hierarchical modelling approach to analyse the presence of *C*. *rodgersii* barrens using a time series of marine imagery collected by an AUV from an east coast Tasmanian NTR and nearby control sites. A survey of barrens inside and outside of the long-established (> 25 year) Governor Island NTR using benthic imagery from an AUV revealed that the NTR has a reduced presence of barrens compared to nearby fished areas [27]; however, analysis was based on a single set of survey data undertaken in 2010–11. Subsequent AUV surveys have been conducted across the same sites, providing data to examine changes through time. The analysis of this novel dataset of marine imagery examines: (i) the difference in presence of urchin barrens inside the NTR compared to control sites (i.e., the “protection effect”); (ii) whether the NTR mitigates the rate of barrens expansion through time; (iii) whether depth and rugosity are important ecological predictors of the presence of barrens; and (iv) whether including spatial and temporal correlation structures improves predictive capability.

## Materials and methods

### Data collection

AUV imagery was collected by the AUV *Sirius*, operated by the University of Sydney’s Australian Centre for Field Robotics as part of the Australian government national IMOS monitoring program. Survey planning and implementation was undertaken by the Institute of Marine and Antarctic Studies (IMAS) at the University of Tasmania (UTas). IMAS operates under a joint-venture between the Tasmanian Government and UTas as the de facto marine research agency. Under this arrangement permits are not required to undertake non-intrusive research (such as conducted here) in state waters, including marine reserves.

The AUV navigates pre-determined transects, with onboard sensors allowing the AUV to maintain a relatively constant altitude of ~ 2m above the seafloor. This results in a predictably stable image footprint of approximately 1.2 X 1.6 m, with imagery captured at a resolution of ~1 mm/pixel.

**Fig 1:**
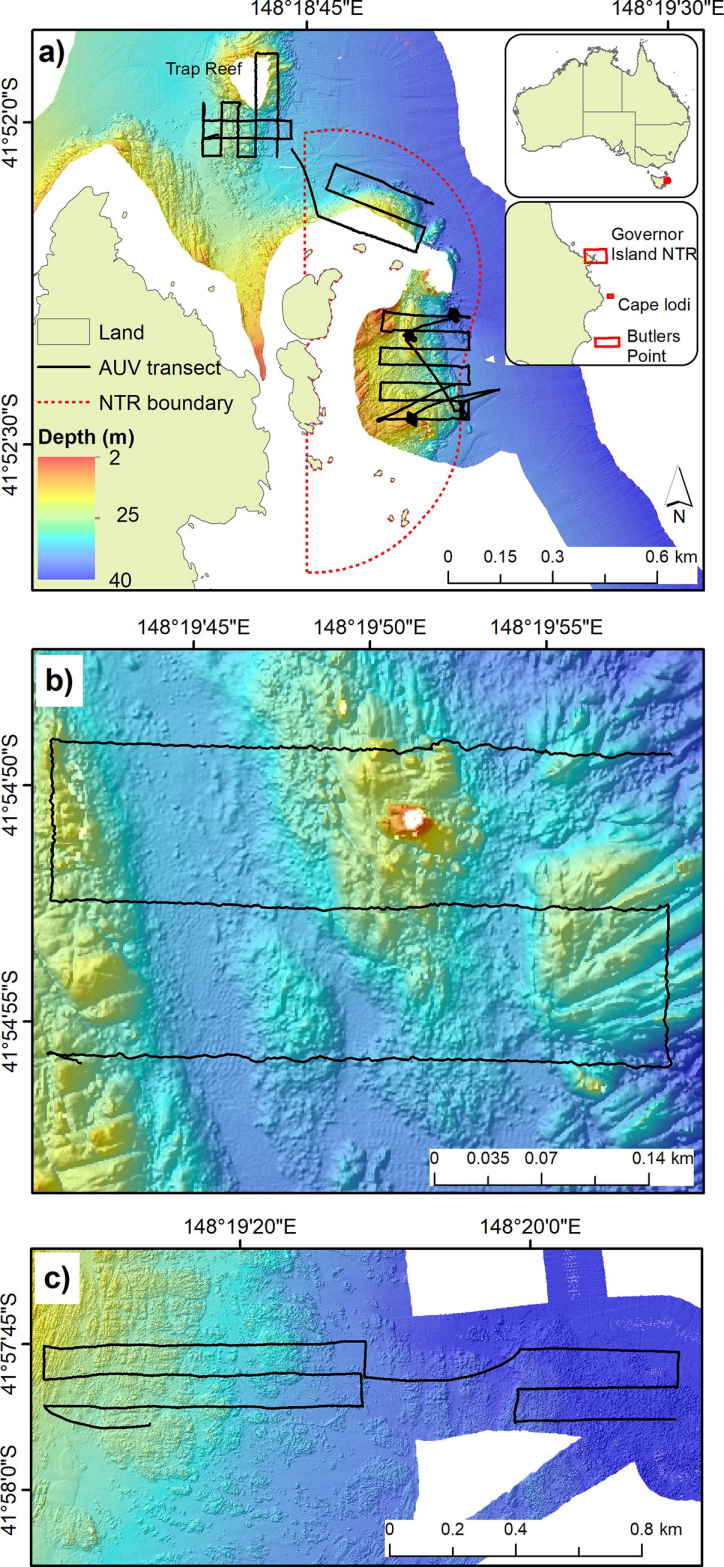
Location of sites used in the study. (A) Governor Island NTR and Trap Reef with inset showing study region; (B) Cape Lodi; and (C) Butlers Point. Underlying bathymetric mapping layer shown in colour. Black lines show locations of repeated AUV transects. All sites were surveyed by AUV in 2011, 2013 and 2016; the Governor Island NTR was also surveyed in 2014. Underlying base data (land and NTR boundary) is from theLIST © State of Tasmania, and supplied under Creative Commons Attribution 3.0 Australia (CC BY 3.0 AU).

Repeated AUV transects were conducted across the study region between 2011 and 2016 (Fig 1). Here, “transect” refers to the complete grid pattern (black lines in Fig 1), with the same pattern repeated each year. Transect locations were approximately centred on: Trap Reef (41°52'01.2"S, 148°18'36.0"E), Governor Island NTR (41°52'22.8"S, 148°18'50.4"E), Cape Lodi (41°54'57.6"S, 148°19'48.0"E) and Butlers Point (41°57'43.2"S, 148°19'01.2"E). All sites were surveyed in 2011, 2013 and 2016. The Governor Island NTR was also surveyed in 2014.

### Rugosity from multibeam sonar bathymetric mapping

*C*. *rodgersii* density is known to be highly correlated with habitat complexity (e.g. [18, 28]). We therefore expected a priori that rugosity, a measure of reef complexity, may be an important covariate to control for when analysing the presence of barrens. Our study region was mapped prior to this study in 2008/09 using multibeam acoustics as part of ongoing mapping by the National Environment and Science Research Program Marine Biodiversity Hub [29]. In order to generate a rugosity measure to use as a model covariate, a 1 metre resolution bathymetric layer from the multibeam sonar was used with the vector rugosity measure (VRM) tool in the Benthic Terrain Modeler 3.0 add-on in ArcGIS 10.6 software. This tool uses vector analysis to calculate rugosity as the variation in the three-dimensional orientation of cells within a specified neighbourhood. We used a neighbourhood of three cells (i.e. three metres) as urchins are likely to respond to complexity on smaller scales. As we expected rugosity to be an important predictor, we excluded the small amount of survey area covered by the AUV transects that did not have underlying multibeam mapping (see Fig 1).

### Image scoring

Scoring followed the methods outlined in Perkins, Hill [27], where individual images were classified as barrens when greater than 50% of the image was bare-rock barren and *C*. *rodgersii* urchins were seen to be either present in the image or in an immediately preceding or succeeding image that was also more than 50% bare-rock. The 50% cut-off was estimated visually by the scorer. For subsequent analysis, an urchin barren was considered either ‘present’ or ‘absent’ at the image level based on this scoring. Urchins, and urchin barrens, are restricted to rocky reef (e.g., [20]) and therefore we restricted our analysis to reef with all images that contained greater than 50% sand excluded. Each scored transect was then subsetted to every fifth image. The subsetting of images was necessary as AUV images overlap and it was determined that every fifth image allowed minimal or no overlap of images for further analysis. This subset of imagery resulted in a total of 20 602 images in the final time-series of data for analysis.

### The models

Statistical analysis of imagery collected along temporally repeated transects at monitoring sites needs to account for the possibility of spatial and temporal correlation in the ecological response data. Failure to account for spatial and/or temporal correlation can lead to biases in model coefficients and confound subsequent statistical inference, often leading to underestimation of residual variance and erroneous conclusions regarding the importance of covariates [30, 31]. To test for the importance of spatio-temporal dependence, three models were fit: 1) model *M*_1_ with neither spatial nor temporal dependence, 2) model *M*_2_ with only spatial dependence, and 3) model *M*_3_ with both spatial and temporal dependence. All models included the same covariates (depth and rugosity) and were nested within the most complex model, *M*_3_, which is described as a Bernoulli separable space-time model:

$$y(s_{i},t)∼Bernoulli(p(s_{i},t))$$ (1)

$$\log\left(\frac{p(s_{i},t)}{1-p(s_{i},t)}\right)=X(s_{i},t)\beta +z(s_{i},t)$$ (2)

$$z(s_{i},t)∼\phi z(s_{i},t-1)+\omega (s_{i},t),t=2,3,\ldots$$ (3)

$$\omega (s,t)∼N(0,\sum (s,\rho ,\sigma^{2}))$$ (4)

$$z(s,1)∼N(0,\sum (s,\rho ,\sigma^{2})/(1-\phi^{2}))$$ (5)

$$\log\rho ∼N(a_{\rho },b_{\rho }^{2})$$ (6)

$$\log\frac{e^{\phi }+1}{e^{\phi }-1}∼N(a_{\phi },b_{\phi }^{2})$$ (7)

$$\log\sigma ∼N(a_{\sigma },b_{\sigma }^{2})$$ (8)

β∼N(0,s) (9)

with *y*(*s*_*i*_,*t*) the categorisation of barren presence for an image located at *s*_*i*_ in year *t*, space and time-varying covariate matrix *X*(*s*_*i*_,*t*) and separable spatio-temporal random effects *z*(*s*,*t*). The temporal covariance function is a stationary autoregressive process, such that −1<*ϕ*<1, where *z*(*s*,*t*) = *ϕz*(*s*,*t*−1)+*ω*(*s*,*t*) with *z*(*s*,1) drawn from the stationary distribution.

The spatial random effects in year *t* have a stationary spatial covariance function with spatial correlation function *H*(*s*−*s*′,*ρ*) for a site *s* and *s*′. We used the Integrated Nested Laplace Approximation approach (INLA; see [32]) for Bayesian spatial and spatio-temporal modelling. The spatial range for INLA is defined by Lindgren, Rue [33] as the distance at which the spatial correlation drops to close to 0.1. The internal parameterisation of the range *ρ* and spatial variance *σ*^2^ used by INLA is given in S1 File. Details of the Bayesian prior specification used in the analysis are given in S2 File. All statistical analyses were conducted within the R statistical computing package [34].

The model covariates for which *β* coefficients were estimated included fixed effects for the NTR (binary, whether an image was located inside the NTR or not), year, an interaction term between NTR and year, depth, depth-squared (to capture expected non-linear effects in deep and shallow images), and rugosity (see above). The model thus allowed for a linear temporal trend in urchin barrens presence over the six-year study duration including a possible interaction with the NTR. Lagged temporal effects and spatial heterogeneity may introduce spatial and temporal dependence on top of the linear trend. The potential impact of spatial and temporal dependence was assessed by comparing model *M*_3_ against the simpler models *M*_1_ and *M*_2_ that dropped these dependence components, as described below. Depth was included as it has been previously found to be an important predictor of barrens presence [20, 27].

Model *M*_2_ is obtained from *M*_3_ by omitting the temporal dependence component (i.e., setting the temporal dependence parameter to zero). Model *M*_1_ is then obtained from model *M*_2_ by also omitting the spatial dependence component (i.e., setting the spatial variance parameter to zero). The hypothesised models are considered equally likely a priori and compared on the basis of the marginal likelihoods given the observed data. The marginal likelihood (or evidence) of each model is proportional to the posterior model probabilities given the a priori equal model weights.

As the values of rugosity were right-skewed (i.e. mostly small values, with a few larger values), to avoid leverage issues a logit transformation was applied to the raw rugosity values. All physical model covariates (i.e. depth, depth-squared and logit-transformed rugosity) were centred by their mean and scaled by their standard deviation to ease comparison of estimated coefficients.

We examined both the percentage change in odds ratios and predicted changes in the probability of urchin barren presence to interpret the relationships with respect to model covariates. For the percentage change in odds ratios given by an increase of one unit in the *i*^th^ covariate, we used the formula (exp(*β*_*i*_)−1)*100. We examined the conditional influence of covariate effects by predicting the posterior probability of barrens presence over the range of values for that covariate in our sample space while holding all other covariates constant and excluding random effects (i.e., conditioning on *z*(*s*′,*t*′) = 0 for predictions to a site *s*’ and year *t*’). The changing probability of barrens presence through time in sites (images) inside and outside the NTR conditioned on the mean values for rugosity and depth inside and outside the NTR respectively. The conditional relationships for depth and rugosity were conditioned on the year 2016 (i.e., the last year surveyed) inside the NTR and were conditioned on the mean values of the alternative covariate inside the NTR. This was accomplished by taking 5000 joint posterior draws of the unknown *β* coefficients from the fitted model.

Code used to fit each model, take posterior samples and explore covariate importance, and the accompanying data is available at: https://metadata.imas.utas.edu.au/geonetwork/srv/en/metadata.show?uuid=d29fa59e-203f-42a8-b0a7-cf77fde7b88a.

## Results

### Data description

**Fig 2:**
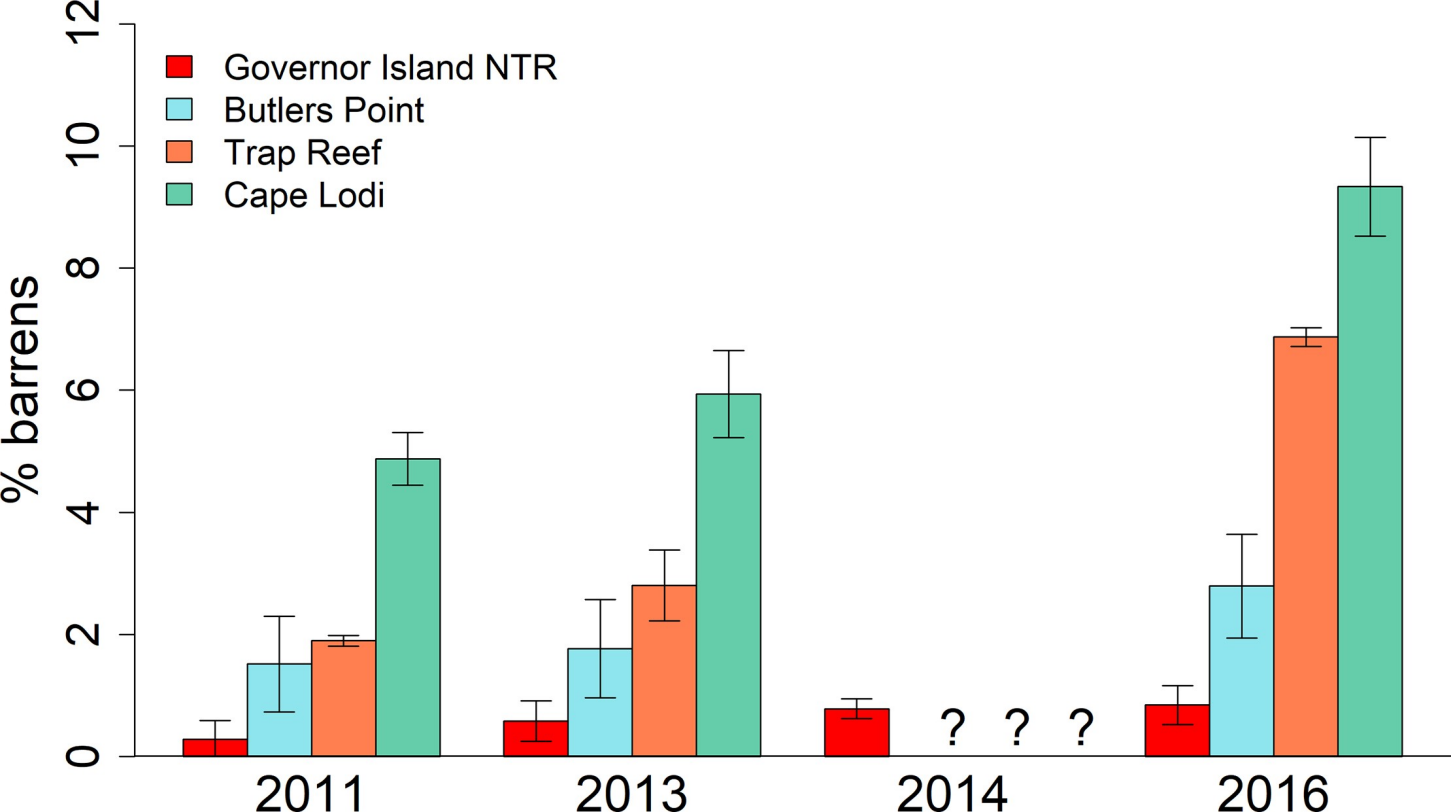
Percent barrens at each of the sites through time. Percent barrens were calculated as the precent of total images scored that were classified as barrens. Note that the control sites were not surveyed in 2014. Error bars are standard errors. Question marks indicate that no data was collected in 2014 for the control sites.

The percent of images classified as barrens showed a general increase through time for all sites (Fig 2). There were distinct differences in the percent barrens at each site, with these differences being maintained through time. The Governor Island reserve (all transects within the NTR pooled within each year surveyed) always had a lower observed percentage of barrens when compared to any of the reference sites, with less than one percent of images being classified as barrens habitat in any year. For the reference sites, Cape Lodi consistently had the highest percent barrens through time, followed by Trap Reef and Butlers Point.

### Model results

Of the three model specifications fitted, the full spatio-temporal model provided the best fit as determined by the model marginal log-likelihoods (S1 Table) and hence also the posterior model probabilities. The INLA posterior model summary for the spatio-temporal model is presented in Table 1. Posterior density plots for all model parameters are provided in S1 Fig.

**Table 1:** INLA model posterior summary for fixed and random effects for the full spatio-temporal model (*M*_3_).

	mean	sd	0.025 quantile	0.5 quantile	0.975 quantile
			**Fixed effects**		
intercept	-7.478	0.353	-8.180	-7.475	-6.793
NTR	-3.150	0.971	-5.180	-3.107	-1.363
year	0.277	0.072	0.137	0.277	0.420
rugosity	0.557	0.105	0.351	0.557	0.765
depth	-0.278	0.242	-0.747	-0.280	0.204
depth-squared	-0.985	0.236	-1.477	-0.975	-0.551
NTR:year	0.070	0.195	-0.300	0.065	0.468
			**Random effects**		
Range (m)	17.055	2.093	13.293	16.938	21.501
Spatial standard deviation	3.413	0.241	2.972	3.401	3.921
Temporal correlation	0.734	0.053	0.621	0.737	0.827

In the full spatial and temporal model (*M*_3_), we found a strong effect of the NTR on the presence of urchin barrens, with the odds of barrens presence being doubled outside of the NTR compared to within the NTR. For an NTR effect, the median coefficient estimate of -3.107 (Table 1, 0.5 quantile) on the logit scale is equivalent to a multiplicative effect on the odds-ratio scale of 0.045 (i.e., exp(-3.107) = 0.045). Therefore, if other model factors are held fixed, the percentage change in the odds of an image being classified as a barren is (exp(-3.107) - 1)x100 = -96% when moving from a site outside of the NTR to within the NTR.

**Fig 3:**
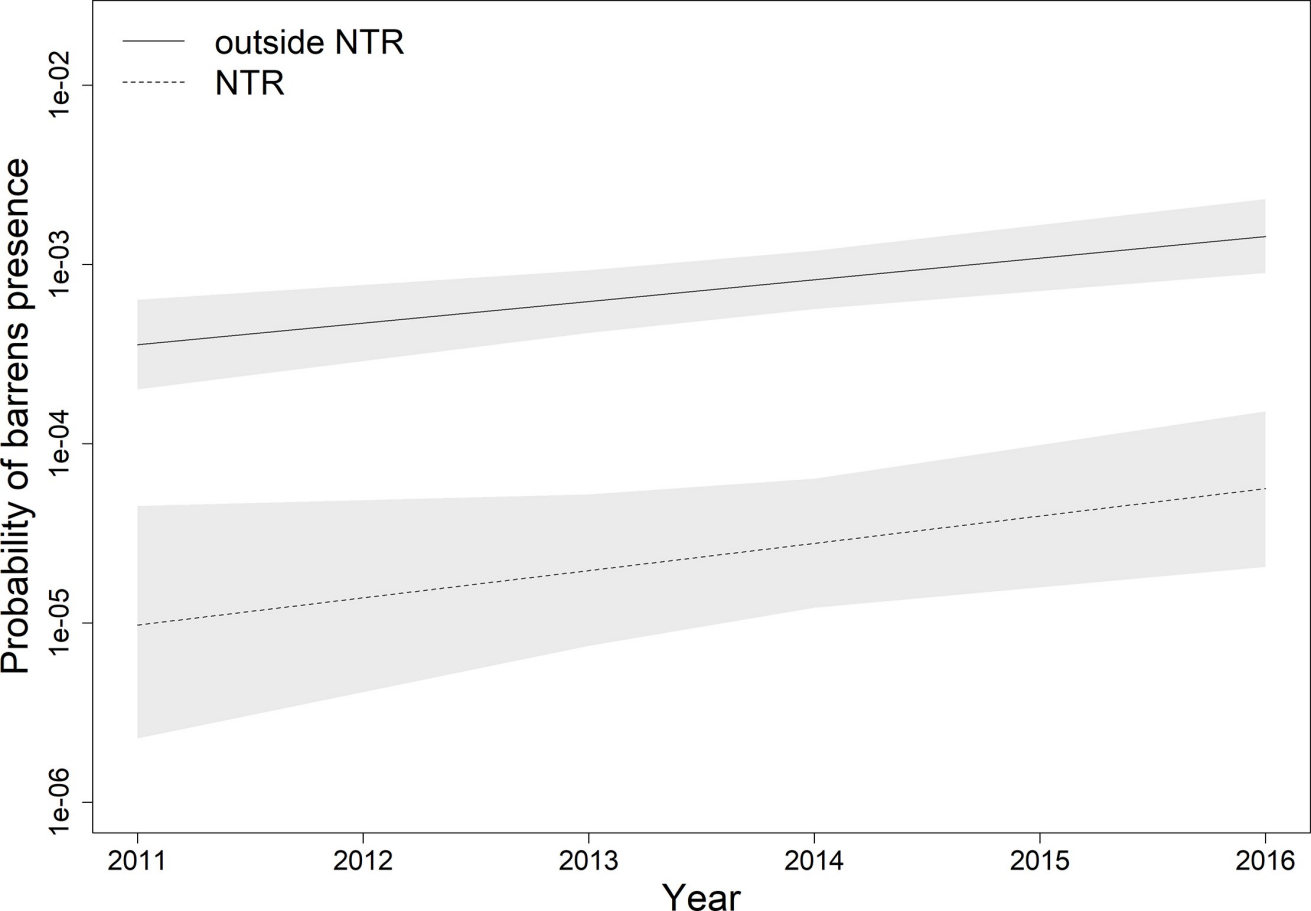
Conditional relationships for probability of barrens presence over the survey period for sites inside and outside the No-Take Reserve (NTR). Probabilities were calculated by taking 5000 posterior draws from the fitted spatio-temporal model (model *M*_3_) and conditioned on the mean values for depth and rugosity from inside and outside the NTR. Predictions were conditioned on spatio-temporal random effects set equal to zero. Lines are posterior means and shaded areas 95% credible intervals. Note logarithmic y-axis.

Over the five-year time period, there was a substantial overall increase in the presence of barrens across all the sites (positive year effect in Table 1 and Fig 3). The median (0.5 quantile) posterior estimate of the coefficient for the year effect was 0.277 (Table 1), which equates to a change in the odds-ratio of barrens presence of 1.319. This means that, when holding all other model factors fixed, the overall percentage increase in the odds of the presence of barrens each year is increasing by 31.9%. However, the rate of change inside the NTR was not found to be substantially different to the reference sites (95% central CI for the NTR × year interaction spans zero in Table 1 and Fig 3). Therefore, there was a rapid and substantial rate of increase in barrens across the region of coastline in this study, but the rate of change inside the NTR was not statistically distinguishable from that of the reference sites (Fig 3).

**Fig 4:**
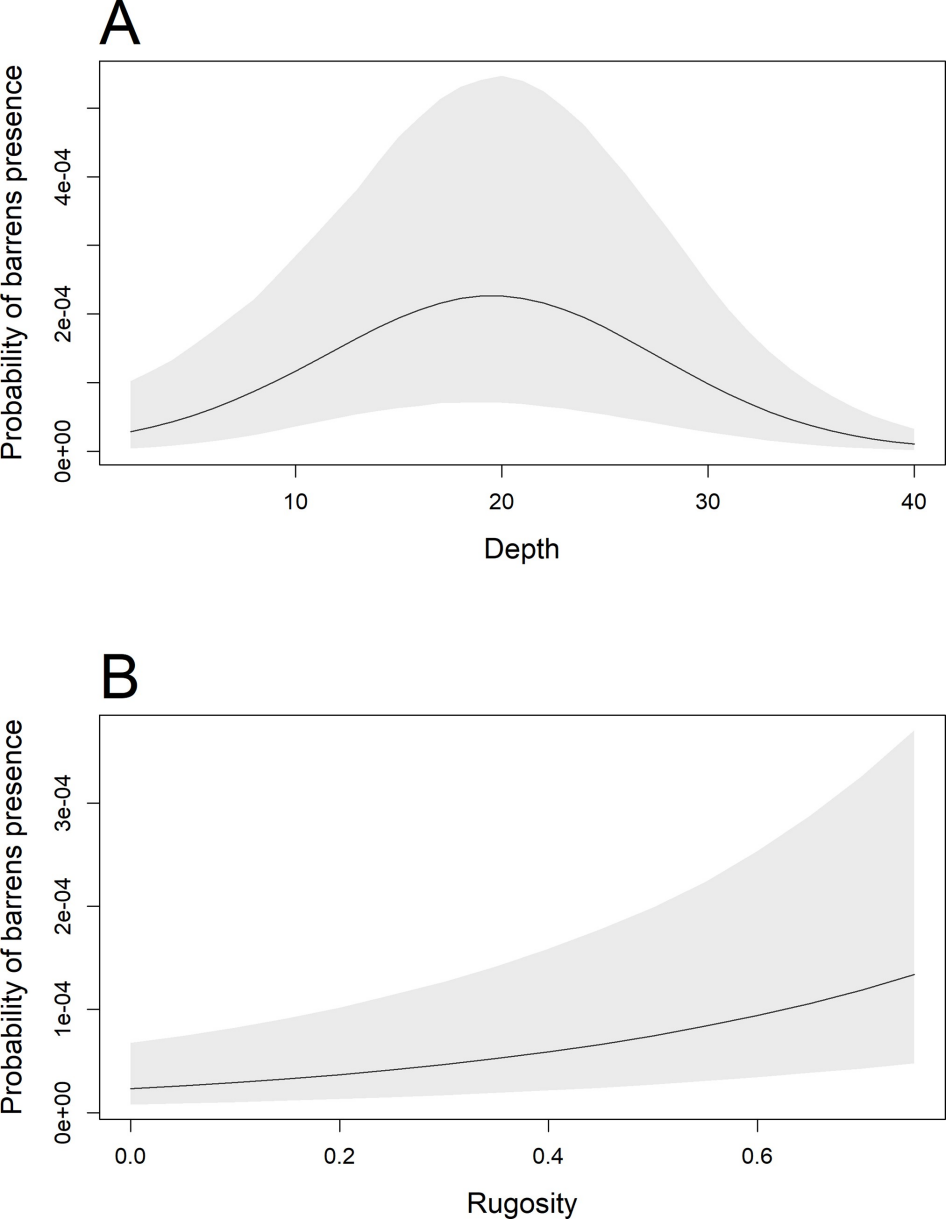
Conditional relationships for probability of barrens presence with respect to (A) depth and (B) rugosity. Probabilities were calculated by taking 5000 posterior draws from the fitted spatio-temporal model (model *M*_3_) and holding all other fixed effects constant while exploring a range of values for the relevant covariate in the absence of random effects. Predictions were conditioned on spatio-temporal random effects set equal to zero. Lines are posterior means and shaded areas 95% credible intervals. We chose the no-take reserve (NTR) in 2016 to calculate the predicted probability. The mean value in the NTR for rugosity was used when exploring depth, and the mean value for depth and depth-squared in the NTR was used when exploring different rugosity values.

After accounting for spatial and temporal dependence, decisive effects were found for the environmental covariates of depth-squared and rugosity (Table 1 and Fig 4). The empirical distribution of barrens across depth in the survey region showed barrens ranged between 15 and 37 m (S2 Fig). The strong effect of depth-squared (Table 1) indicates a concave relationship between urchin barrens presence and depth. For a hypothetical site within the NTR in the year 2016 with mean rugosity, the conditional relationship between depth and the probability of barrens presence is plotted in Fig 4A. The peak probability for urchin barrens presence is approximately 20 m, with low probability beyond 40 m. Thus, there is a quadratic effect of depth, with an overall lower presence of barrens in the shallower and deeper images collected (Fig 4A).

For a hypothetical site within the NTR in the year 2016, but now at a constant mean depth with rugosity varying, the conditional relationship between and the presence of barrens was positive (Fig 4B). Rugosity values in the raw data ranged from near zero to 0.544, with a mean of 0.015 ± 0.030 inside the NTR and a mean of 0.011 ± 0.018 at the reference sites. Rugosity values of unsampled bathymetric cells in the region ranged up to 0.758.

Model comparisons showed that both spatial and temporal correlation were important in explaining the presence of urchin barrens overall (S1 Table). Results for the spatial random effects showed that spatial correlation occurred over a mean range of approximately 17 m (Table 1 and S1 Fig) with mean spatial standard deviation of 3.413 (Table 1 and S1 Fig). The posterior distribution of the AR1 temporal correlation parameter was found to have a mean of 0.734 ± 0.053, implying a strong temporal correlation effect for the presence of barrens with barrens status likely to persist throughout the time period.

## Discussion

Here we utilize marine imagery collected from an AUV to demonstrate that an NTR has a substantial localised effect on the formation of barrens by a range extending urchin species in a region that is experiencing rapid climate change. We find a doubling in the odds of the presence of barrens in reference sites outside the NTR; however, although the NTR had consistently lower presence of barrens through time, we also find that the data collected to date does not indicate that the rate of increase in barrens inside the NTR is different to that outside. Therefore, our results imply that the NTR may have conferred resilience in terms of the resistence to the initial establishment of barrens, but that there is currently no evidence of recovery inside the NTR once barrens are established. Preventing the phase-shift from healthy rocky reef ecosystems to large areas of urchin barrens that have occurred elsewhere on the east coast of Tasmania is likely to require the ongoing maintenance of low densities of urchins [22]. Therefore, continued monitoring of this study region is necessary to establish whether the NTR maintains a lower prevalence of barrens or a differing rate of barrens expansion. Monitoring data could also be used to set trigger points for management responses such as the spatial targeting of an emerging urchin fishery (see [35]).

Our results regarding the rate of increase in barrens through time are in concurrence with other recent findings that demonstrate an alarming expansion of *C*. *rodgersii* barrens in this section of coastline [18]. Ling and Keane [18] found that barrens were not detected in SCUBA and towed video surveys in this region in 2001/2, but were approximately 2% of surveyed habitat in 2016/17. We found that over our survey period the median probability of any given image being classified as an urchin barren was increasing by 31.9% per year. While the proportion of barrens habitat was still lower than 10% for any of our sites in 2016, the rapid increase in barrens over the 5 year period provides a concerning trajectory. Robust monitoring programs such as the current AUV program are necessary to inform the effective adaptive management of nearshore habitats and economically important fisheries that depend upon them.

We demonstrate that a rugosity measure derived from multibeam data and a quadratic depth term are both strongly correlated with urchin barren presence and therefore may aid in the fine-scale prediction of areas likely to see ongoing increases in the presence of barrens habitat. This information could be used in the spatial planning of target areas for conservation efforts and monitoring [36]. Our research demonstrates that imagery collected by AUVs provides a benthic monitoring platform capable of tracking important ecological changes thereby providing important and timely information for the management of the marine environment.

### Resilience and protection effects

Resilience can be framed in terms of a systems ability to both resist and recover from disturbance (see [4]), with disturbances pushing systems towards alternative stable states. Urchin barrens represent an alternative stable state in our study system, with high densities of urchins capable of overgrazing kelp, eventually pushing the system to an alternate state of widespread barrens. *C*. *rodgersii* urchin barrens are known to demonstrate a hysteresis, whereby the level of urchin density required to return reefs to productive kelp beds is much lower than that required to result in the phase shift to barrens [22]. In areas of the east coast of Tasmania where urchins settled earlier, widespread barrens are now common [18]. Therefore, understanding whether conservation measures such as NTRs can affect the ability of communities to both resist and recover from the establishment of *C*. *rodgersii* urchins is important.

Our results clearly demonstrate the resistance to the establishment of *C*. *rodgersii* urchin barrens of a Tasmanian NTR when compared to nearby reference areas where fishing is permitted. While our study is limited to a single NTR, the findings are supported by a study examining a time series of SCUBA monitoring of NTR and reference sites across Tasmania that found that areas protected from fishing activities resisted the colonisation by *C*. *rodgersii* and facilitated greater cover of macroalgae [7]. This suggests that NTRs offer a spatial management tool to aid in limiting the expansion of barrens habitat on the east coast of Tasmania. While our study does not test the causal relationship for the lower presence, the higher density of the rock lobster *J*. *edwardsii* (and especially large lobsters) inside Tasmanian NTRs [37–39] and the higher predation rates of urchins in those reserves by lobsters [21] suggests that urchin densities are likely to be kept lower in the NTR through higher predation rates. The Governor Island NTR, established in 1991, was likely to have had a much higher density and larger size structure of lobsters than adjacent coastal waters when *C*. *rodgersii* urchins began arriving in this area in significant numbers during the 1990’s [20, 37]. As *J*. *edwardsii* also shows high site fidelity [40], higher localised predation rates within the reserve are likely to have maintained lower levels of barrens.

Although our analysis demonstrates a strong protection effect in terms of resistance to the establishment of barrens, the data collected to date does not demonstrate that recovery is occurring inside the NTR. In fact, current rates of increase in barrens were not distinguishable between the NTR and reference sites, suggesting that habitat suitable for barrens formation in both are being pushed towards alternative stable states. The high temporal correlation in the presence of barrens is in agreeance with other studies that show that, once *C*. *rodgersii urchins* are established, recovery of kelp habitat is problematic [22]. Whether the proportion of barrens habitat can be restricted to a level which prevents catastrophic phase shifts to an alternative stable state [22], or perhaps even promotes a recovery, requires ongoing monitoring and adaptive management. Monitoring approaches such as the AUV-based program reported here provide a useful platform for ongoing work and to test ideas around the resilience of NTRs to the effects of climate change.

### The importance of rugosity and depth for the presence of barrens

Habitat complexity is an important factor in the distribution of many species and if quantified adequately over large areas may provide a means to better predict species distributions. For *C*. *rodgersii* urchins, the importance of habitat complexity that provides refuge for urchins has been demonstrated in diver-based studies (e.g., [18, 20]). Our results demonstrate that a measure of habitat complexity derived from bathymetric mapping provides an important covariate for barrens presence and thus could be a useful predictor over large areas. Further exploration of the use of multibeam mapping derived complexity measures and the optimal scale at which to quantify complexity for *C*. *rodgersii* would help identify areas that should receive targeted efforts to control the expansion of urchin barrens habitat. For example, predictive maps that incorporate habitat complexity could be used to better understand the likely future extent and spatial distribution of barren formation, and inform adaptive management responses, such as a subsidised urchin fishery (see [35]).

Our findings regarding the depth distribution of urchin barrens in Tasmania agrees with previous research that shows that while barrens do extend into deeper areas of reef (> 40 metres), the majority of barrens are expected to form in 10–30 metres [18, 20, 27]. The strong negative coefficient for depth-squared indicates a negative quadratic relationship of barrens presence with depth. Therefore, our results are in agreeance with ecological expectations of the distribution of barrens across our study sites, with lower presence in both deep and shallow images.

### The modelling approach

The Bayesian hierarchical approach used here provides a means of modelling the time-series of benthic imagery that captures inherent spatial and temporal structures in the data. We found that including both spatial and spatio-temporal dependence improved model fits to the data. Spatial dependence was found to occur over relatively short distances (mean of approximately 17 m), with relatively high spatial variance. This reflects the patchy nature of barrens in the early stages, where smaller “incipient” barrens form as early arrivals settle in areas where complex habitat allows the establishment of discrete barrens patches.

Previous research using the same sites but just a single timepoint, and aggregating data into transects found a suggestive but non-significant effect of the NTR on the presence of barrens [27]. Here, rather than aggregating the data, we use images as the basis of the analysis. Increasing the sample size to a large number of images rather than a few transects increases the probability of detecting a temporal change in urchin barren presence. Our analysis showed that spatio-temporal dependence is an important feature of the imagery data that should be accounted for by statistical modelling. We therefore advocate for the use of similar approaches in future analysis of marine imagery datasets generated by ecological monitoring programs.

## Conclusions

We demonstrate that a time-series of imagery from an AUV was able to detect a strong and consistent protection effect on the presence of urchin barrens in a study region on the east coast of Tasmania. Although the NTR has lower rates of barrens presence we also found the odds of barrens presence increasing at approximately 31.9% per year in both NTR and reference sites. The spatio-temporal dependence revealed by the modelling further suggests that urchin barrens, once established, persist through time. Therefore, there is currently strong evidence for the resistance of the NTR to barrens formation, but importantly there is also no evidence of recovery. While barrens cover is currently relatively low inside the NTR, a slowing of the rate of increase or a recovery of kelp habitat may be necessary to prevent a widespread shift to persistent barrens. With ongoing climate change related warming and incursion of urchins into new areas, monitoring the increase in barrens is critical to informing adaptive management and developing innovative solutions. To support these efforts, we also report that measures of reef complexity and depth from multibeam sonar mapping provide important predictors for the occurrence of barrens. Therefore, detailed seabed mapping and spatial analysis may help in spatial management when targeting efforts to reduce urchin numbers via mechanisms such as spatially targeted fishing incentives for urchin divers. Finally, we advocate the use of spatio-temporal models such as those presented here when assessing time-series of benthic imagery due to their flexible nature and ability to incorporate spatial and temporal correlation inherent in imagery data.

## Supporting information

S1 File Internal parameterisation of range and spatial variance used by INLA. (DOCX)

S2 File Prior specification. (DOCX)

S1 Table Model posterior summaries for non-spatial, spatial and the full model presented in the main text including marginal log-likelihoods. (DOCX)

S1 Fig Model posterior densities for fixed (A) and random (B) effects.
(TIF)

S2 Fig Percent barrens by depth over the entire time-series across all sites. Percent barrens was calculated as the percentage of images within each one metre depth bin that was classified as barrens across all images scored. The number of images scored across depth (black line) is included to show the range of depths sampled and the intensity of sampling with depth.
(TIF)

S3 Fig Percent barrens by depth for 2011, 2013 and 2016 across all sites. Percent barrens was calculated as the percentage of images within each one metre depth bin that was classified as barrens across all images scored within that year. The number of images scored across depth (black line) is included to show the range of depths sampled and the intensity of sampling with depth. 2014 was not included as only the NTR was sampled in that year.
(TIF)

## References

[1] Poloczanska ES, Brown CJ, Sydeman WJ, Kiessling W, Schoeman DS, Moore PJ, et al Global imprint of climate change on marine life. Nature Climate Change. 2013;3(10):919–25.

[2] Perry AL, Low PJ, Ellis JR, Reynolds JD. Climate change and distribution shifts in marine fishes. Science. 2005;308(5730):1912–5. 10.1126/science.1111322 15890845

[3] Holling CS. Resilience and Stability of Ecological Systems. Annual Review of Ecology and Systematics. 1973;4(1):1–23.

[4] Hodgson D, McDonald JL, Hosken DJ. What do you mean, 'resilient'? Trends in ecology & evolution. 2015;30(9):503–6. 26159084 10.1016/j.tree.2015.06.010

[5] Bates AE, Cooke RSC, Duncan MI, Edgar GJ, Bruno JF, Benedetti-Cecchi L, et al Climate resilience in marine protected areas and the ‘Protection Paradox’. Biol Conserv. 2019;236:305–14.

[6] Carpenter S, Walker B, Anderies JM, Abel N. From Metaphor to Measurement: Resilience of What to What? Ecosystems. 2001;4:765–81.

[7] Bates AE, Stuart-Smith RD, Barrett NS, Edgar GJ. Biological interactions both facilitate and resist climate-related functional change in temperate reef communities. Proceedings Biological sciences / The Royal Society. 2017;284(1856). 10.1098/rspb.2017.0484 PMC5474073 28592671

[8] Bates AE, Barrett NS, Stuart-Smith RD, Holbrook NJ, Thompson PA, Edgar GJ. Resilience and signatures of tropicalization in protected reef fish communities. Nature Climate Change. 2013;4(1):62–7.

[9] Mellin C, Aaron MacNeil M, Cheal AJ, Emslie MJ, Julian Caley M. Marine protected areas increase resilience among coral reef communities. Ecol Lett. 2016;19(6):629–37. 10.1111/ele.12598 27038889

[10] Strain EMA, Edgar GJ, Ceccarelli D, Stuart-Smith RD, Hosack GR, Thomson RJ, et al A global assessment of the direct and indirect benefits of marine protected areas for coral reef conservation. Divers Distrib. 2019;25(1):9–20.

[11] Vanhatalo J, Hosack GR, Sweatman H. Spatiotemporal modelling of crown-of-thorns starfish outbreaks on the Great Barrier Reef to inform control strategies. J Appl Ecol. 2017;54(1):188–97.

[12] Worm B, Barbier EB, Beaumont N, Duffy JE, Folke C, Halpern BS, et al Impacts of biodiversity loss on ocean ecosystem services. Science. 2006;314(5800):787–90. 10.1126/science.1132294 17082450

[13] Byrnes JE, Reynolds PL, Stachowicz JJ. Invasions and extinctions reshape coastal marine food webs. Plos One. 2007;2(3):e295 10.1371/journal.pone.0000295 17356703 PMC1808429

[14] Bulleri F, Tamburello L, Benedetti-Cecchi L. Loss of consumers alters the effects of resident assemblages on the local spread of an introduced macroalga. Oikos. 2009;118(2):269–79.

[15] Ridgeway KR. Long-term trend and decadal variability of the southward penetration of the East Australian Current. Geophysical Research Letters. 2007;34(L13613).

[16] Dunstan PK, Foster SD, King E, Risbey J, O'Kane TJ, Monselesan D, et al Global patterns of change and variation in sea surface temperature and chlorophyll a. Sci Rep. 2018;8(1):14624 10.1038/s41598-018-33057-y 30279444 PMC6168485

[17] Robinson LM, Gledhill DC, Moltschaniwskyj NA, Hobday AJ, Frusher S, Barrett N, et al Rapid assessment of an ocean warming hotspot reveals "high" confidence in potential species' range extensions. Global Environmental Change-Human and Policy Dimensions. 2015;31:28–37.

[18] Ling S, Keane JP. Resurvey of the Longspined Sea Urchin (Centrostephanus rodgersii) and associated barren reef in Tasmania. Hobart, Tasmania: Institute for Marine and Antarctic Studies; 2018.

[19] Ling SD. Range expansion of a habitat-modifying species leads to loss of taxonomic diversity: a new and impoverished reef state. Oecologia. 2008;156(4):883–94. 10.1007/s00442-008-1043-9 18481099

[20] Johnson CR, Ling S, Ross J, Shepherd S, Miller K. Establishment of the long-spined sea urchin (Centrostephanus rodgersii) in Tasmania: first assessment of potential threat to fisheries. Hobart, Tasmania; 2005.

[21] Ling SD, Johnson CR. Marine reserves reduce risk of climate-driven phase shift by reinstating size and habitat-specific trophic interactions. Ecological Applications. 2012;22(4):1232–45. 10.1890/11-1587.1 22827131

[22] Ling SD, Johnson CR, Frusher SD, Ridgway KR. Overfishing reduces resilience of kelp beds to climate-driven catastrophic phase shift. Proceedings of the National Academy of Sciences of the United States of America. 2009;106(52):22341–5. 10.1073/pnas.0907529106 20018706 PMC2793314

[23] Pizarro O, Williams SB, Jakuba MV, Johnson-Roberson M, Mahon I, Bryson M, et al., editors. Benthic monitoring with robotic platforms—the experience of Australia. 2013 IEEE International Underwater Technology Symposium, UT 2013; 2013; Tokyo.

[24] Durden JM, Schoening T, Althaus F, Friedman A, Garcia R, Glover AG, et al Perspectives in Visual Imaging for Marine Biology and Ecology: From Acquisition to Understanding In: Hughes DJH R. N., Smith I. P., and Dale A. C., editor. Oceanography and Marine Biology: An Annual Review, Vol 54 Boca Raton, Florida: CRC Press; 2016.

[25] Hill NA, Lucieer V, Barrett NS, Anderson TJ, Williams SB. Filling the gaps: Predicting the distribution of temperate reef biota using high resolution biological and acoustic data. Estuarine, Coastal and Shelf Science. 2014;147:137–47.

[26] Perkins NR, Hosack GR, Foster SD, Hill NA, Barrett NS. Spatial properties of sessile benthic organisms and the design of repeat visual survey transects. Aquatic Conservation: Marine and Freshwater Ecosystems. 2018;29(1):59–71.

[27] Perkins NR, Hill NA, Foster SD, Barrett NS. Altered niche of an ecologically significant urchin species, Centrostephanus rodgersii, in its extended range revealed using an Autonomous Underwater Vehicle. Estuarine, Coastal and Shelf Science. 2015;155:56–65.

[28] Flukes EB, Johnson CR, Ling SD. Forming sea urchin barrens from the inside out: an alternative pattern of overgrazing. Marine Ecology Progress Series. 2012;464:179–94.

[29] Nichol SL, Anderson TJ, McArthur M, Barrett NS, Heap AD, Siwabessy JPW, et al 2009 Southeast Tasmania temperate reef survey: post survey report. Geoscience Australia; 2009.

[30] Dormann CF. Effects of incorporating spatial autocorrelation into the analysis of species distribution data. Global Ecology and Biogeography. 2007;16(2):129–38.

[31] Legendre P. Spatial Autocorrelation: Trouble or New Paradigm? Ecology. 1993;74(6):1659–73.

[32] Rue H, Martino S, Chopin N. Approximate Bayesian Inference for Latent Gaussian Models by Using Integrated Nested Laplace Approximations. Journal of the Royal Statistical Society. 2009;71(2):319–92.

[33] Lindgren F, Rue H, Lindstrom J. An explicit link between Gaussian fields and Gaussian Markov random fields: the stochastic partial differential equation approach. J Roy Stat Soc B. 2011;73:423–98.

[34] R Core Team. R: A language and environment for statistical computing. R Foundation for Statistical Computing Vienna, Austria 2019.

[35] Cresswell K, Keane JP, Ogier E, Yamazaki S. Centrostephanus Subsidy Program: Initial Evaluation. Hobart, Tasmania: University of Tasmania, Institute for Marine and Antarctic Studies; 2019.

[36] Hayes KR, Hosack GR, Lawrence E, Hedge P, Barrett NS, Przeslawski R, et al Designing Monitoring Programs for Marine Protected Areas Within an Evidence Based Decision Making Paradigm. Frontiers in Marine Science. 2019;6.

[37] Buxton C, Barrett N, Haddon M, Gardner C, Edgar G. Evaluating the effectiveness of Marine Protected Areas as a fisheries management tool. Hobart, Tasmania: Tasmanian Acquaculture and Fisgeries Institute; 2006. Contract No.: FRDC Project No. 1999/162

[38] Barrett NS, Buxton CD, Edgar GJ. Changes in invertebrate and macroalgal populations in Tasmanian marine reserves in the decade following protection. Journal of Experimental Marine Biology and Ecology. 2009;370(1–2):104–19.

[39] Edgar GJ, Barrett NS. Effects of the declaration of marine reserves on Tasmanian reef fishes, invertebrates and plants. Journal of Experimental Marine Biology and Ecology. 1999;242(1):107–44.

[40] Barrett N, Buxton C, Gardner C. Rock lobster movement patterns and population structure within a Tasmanian Marine Protected Area inform fishery and conservation management. Marine and Freshwater Research. 2009;60(5):417–25.

