# Supplementary material for: Monitoring the resilience of a no-take marine reserve to a range extending species using benthic imagery
Source: PLoS One. 2020 Aug 12;15(8):e0237257. doi: 10.1371/journal.pone.0237257 (PMC7423107; doi:10.1371/journal.pone.0237257)
Supplement: S1 File — (DOCX) [file pone.0237257.s001.docx]

**S1**

**Internal parameterisation of range and spatial variance used by INLA**

The internal parameterisation of the range used by INLA is:

|  | $\rho= \sqrt{8\nu}/\kappa$, |  |
| --- | --- | --- |

where $\nu= \alpha-D/2$ is the smoothness parameter of the Matern covariance function, $D=2$ is the dimensionality of the spatial Gaussian field and $\kappa$ is the spatial scale parameter. In actual fact, and one used by the prior specification, is that $\rho\approx0.13$ according to Lindgren and Rue (1), this is true for all $\nu>1/2$. The default adopted by INLA is $\alpha=2$, in which case Blangiardo and Cameletti (2, Ch. 6) point out that, for $\nu=1$,

|  | $\rho= \sqrt{8}/\kappa$ |  |
| --- | --- | --- |
|  | $\sigma^{2}=1/(4\pi\kappa^{2}\tau^{2})$, |  |

where $\tau$ and $\kappa$ correspond to the SPDE parameterisation used internally by INLA.

References

1. Lindgren F, Rue H. Bayesian spatial modelling with R-INLA. J Stat Softw. 2015;63(19):1-25.

2. Blangiardo M, Cameletti M. Spatial and spatio-temporal Bayesian models with R-INLA: Wiley; 2015. 308 p.
