# Supplementary material for: Monitoring the resilience of a no-take marine reserve to a range extending species using benthic imagery
Source: PLoS One. 2020 Aug 12;15(8):e0237257. doi: 10.1371/journal.pone.0237257 (PMC7423107; doi:10.1371/journal.pone.0237257)
Supplement: S2 File — (DOCX) [file pone.0237257.s002.docx]

**S2**

**Prior Specification**

Independent marginal priors are specified for all parameters except the spatio-temporal standard deviation $\sigma$ and spatial range $\rho$, which are given a joint prior as described below.

**Prior for spatio-temporal variance and spatial range**

For $\sigma$ and $\rho$ the penalised complexity prior (pc prior; Simpson et al., 2017) approach was taken. Full details of the approach are outlined in Fuglstad et al. (2018). A joint PC prior corresponding to a base model with infinite range and zero variance is

$$\pi\left( \sigma, \rho\right)= \frac{d}{2}\tilde{\lambda}_{1}\tilde{\lambda}_{2}\rho^{-d/{2-1}}\exp\left( -\tilde{\lambda}_{1}\rho^{{-d}/2}- \tilde{\lambda}_{2}\sigma\right),$$

$$\sigma>0, \rho>0,$$

where $P\left( \rho< \rho_{0} \right)= \alpha_{1}$ and $P\left( \sigma> \sigma_{0} \right)= \alpha_{2}$ are achieved by

$\tilde{\lambda}_{1}= -log(\alpha_{1}){\rho_{0}}^{d/2}$ and $\tilde{\lambda}_{2}= -\frac{log(\alpha_{2})}{\sigma_{0}}$.

The smoothness parameter $d$ was set to a default value of 2. In INLA, the approach taken to specify the $\tilde{\lambda}_{1}$ and $\tilde{\lambda}_{2}$ is through the relationships

$$P\left( \rho< \rho_{0} \right)= p_{\rho}$$

$P\left( \sigma>\sigma_{0} \right)= p_{\sigma}$,

where the lower tail quantile and probability for the range ($\rho_{0}$ and $p_{\rho}$ respectively) and the upper tail quantile and probability for the standard deviation ($\sigma_{0}$ and $p_{\sigma}$ respectively) are specified.

Our study region spanned a distance of approximately 10 km (Fig. 1). However, in the early stages of establishment, barrens tend to be very patchy and “incipient” (see Johnson et al., 2005) in nature. Urchins become established where refuge is available in cracks and crevices, and primarily feed nocturnally, returning to these ‘home’ crevices. The areas of these barrens patches tend to range from 1 to 10 m^2^ (Flukes et al., 2012). We chose to set a weakly informative prior (see Simpson et al., 2017) for the spatial range based on the possibility of correlation occurring only at the scale of a single incipient barren. Thus, we set a pc prior for the range with $\rho_{0}=10$ and $p_{\rho}=0.1$. That is, there was a 10% probability that the spatial range was less than 10 metres.

We also set the prior for the standard deviation by setting $\sigma_{0}=1$ and $p_{\sigma}=0.2$, that is, there is a 20% probability that the standard deviation is > 1.

**Prior for temporal autocorrelation**

The prior for the temporal autocorrelation specifies $P(\phi<0.8)=P(\phi>0.9)=0.2$ where $\phi$is the correlation between two years at a given image location. For INLA, this prior for $\phi$ is given by,

$$\log\left( \frac{1+ \phi}{1- \phi} \right) \sim N(a_{\phi}, b_{\phi}^{2})$$

where $a_{\phi}=\exp(0.95)$ and $b_{\phi}=0.45$.

**Prior for regression coefficients**

We used the default prior for the mean and precision (i.e. the inverse of the variance) of the intercept of $N(0, 0)$. For the regression coefficients we altered the default precision of 0.001 to 0.01, giving priors of $N(0, 0.01)$ for the coefficients of all (transformed and scaled) covariates.
