# Supplementary material for: Monitoring the resilience of a no-take marine reserve to a range extending species using benthic imagery
Source: PLoS One. 2020 Aug 12;15(8):e0237257. doi: 10.1371/journal.pone.0237257 (PMC7423107; doi:10.1371/journal.pone.0237257)
Supplement: S1 Table — (DOCX) [file pone.0237257.s003.docx]

**S3**

Model posterior summaries for non-spatial, spatial and the full model presented in the main text including marginal log-likelihoods.

**Model *M*_1_: non-spatial model**

|  | **mean** | **sd** | **0.025 quantile** | **0.5 quantile** | **0.975 quantile** |
| --- | --- | --- | --- | --- | --- |
|  |  |  | **Fixed effects** |  |  |
| intercept | -3.821 | 0.127 | -4.074 | -3.819 | -3.577 |
| NTR | -2.153 | 0.436 | -3.062 | -2.134 | -1.348 |
| year | 0.203 | 0.026 | 0.154 | 0.203 | 0.254 |
| rugosity | 0.417 | 0.052 | 0.315 | 0.417 | 0.520 |
| depth | 0.117 | 0.089 | -0.055 | 0.116 | 0.295 |
| depth-squared | -0.658 | 0.092 | -0.845 | -0.656 | -0.484 |
| NTR:year | 0.086 | 0.086 | 0.084 | 0.084 | 0.262 |

**Marginal log-Likelihood: -1961.43**

**Model *M*_2_: spatial model**

|  | **mean** | **sd** | **0.025 quantile** | **0.5 quantile** | **0.975 quantile** |
| --- | --- | --- | --- | --- | --- |
|  |  |  | **Fixed effects** |  |  |
| intercept | -6.465 | 0.275 | -7.008 | -6.643 | -5.929 |
| NTR | -2.886 | 0.771 | -4.469 | -2.861 | -1.442 |
| year | 0.219 | 0.040 | 0.141 | 0.219 | 0.299 |
| rugosity | 0.461 | 0.095 | 0.275 | 0.461 | 0.649 |
| depth | -0.349 | 0.229 | -0.794 | -0.351 | 0.104 |
| depth-squared | -0.847 | 0.216 | -1.297 | -0.839 | -0.447 |
| NTR:year | 0.068 | 0.133 | 0.064 | 0.064 | 0.340 |
|  |  |  | **Random effects** |  |  |
| Range (m) | 21.95 | 3.360 | 16.08 | 21.70 | 29.26 |
| Spatial standard deviation | 2.82 | 0.212 | 2.42 | 2.81 | 3.25 |

**Marginal log-Likelihood: -1536.64**

**Model *M*_3_: spatio-temporal model**

|  | **mean** | **sd** | **0.025 quantile** | **0.5 quantile** | **0.975 quantile** |
| --- | --- | --- | --- | --- | --- |
|  |  |  | **Fixed effects** |  |  |
| intercept | -7.478 | 0.353 | -8.180 | -7.475 | -6.793 |
| NTR | -3.150 | 0.971 | -5.180 | -3.107 | -1.363 |
| year | 0.277 | 0.072 | 0.137 | 0.277 | 0.420 |
| rugosity | 0.557 | 0.105 | 0.351 | 0.557 | 0.765 |
| depth | -0.278 | 0.242 | -0.747 | -0.280 | 0.204 |
| depth-squared | -0.985 | 0.236 | -1.477 | -0.975 | -0.551 |
| NTR:year | 0.070 | 0.195 | -0.300 | 0.065 | 0.468 |
|  |  |  | **Random effects** |  |  |
| Range (m) | 17.055 | 2.093 | 13.293 | 16.938 | 21.501 |
| Spatial standard deviation | 3.413 | 0.241 | 2.972 | 3.401 | 3.921 |
| Temporal correlation | 0.734 | 0.053 | 0.621 | 0.737 | 0.827 |

**Marginal log-Likelihood: -1481.52**
