# Supplementary figures and images for: Monitoring the resilience of a no-take marine reserve to a range extending species using benthic imagery
Source: PLoS One. 2020 Aug 12;15(8):e0237257. doi: 10.1371/journal.pone.0237257 (PMC7423107; doi:10.1371/journal.pone.0237257)

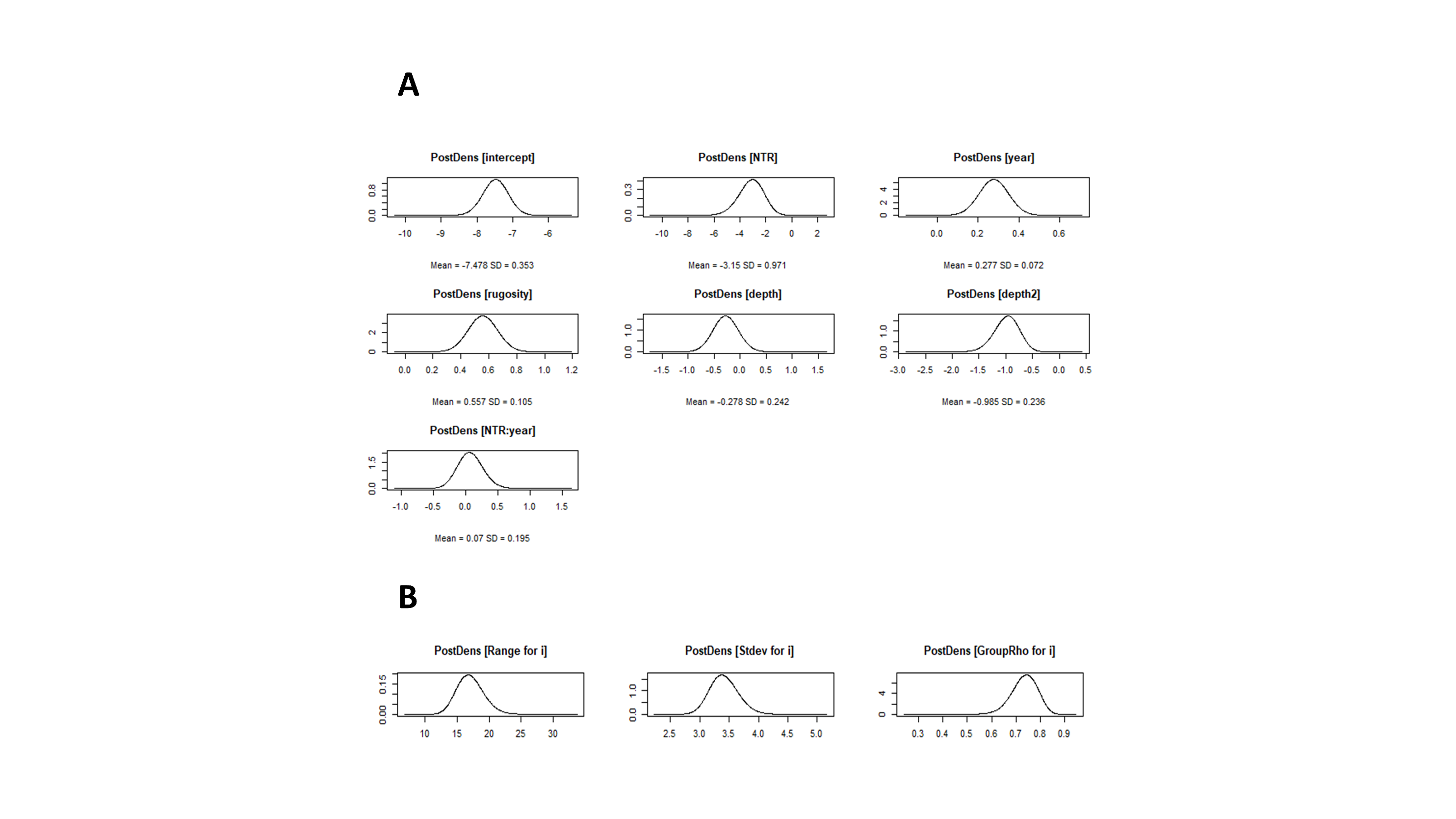

Supplement: S1 Fig — Model posterior densities for fixed (A) and random (B) effects. (TIF) [file pone.0237257.s004.tif]
